# Supplementary material for: A high-throughput screening and computation platform for identifying synthetic promoters with enhanced cell-state specificity (SPECS)
Source: Nat Commun. 2019 Jun 28;10:2880. doi: 10.1038/s41467-019-10912-8 (PMC6599391; doi:10.1038/s41467-019-10912-8)
Supplement: Supplementary file 1 — Supplementary Information [file 41467_2019_10912_MOESM1_ESM.pdf]

A High-Throughput Screening and Computation Platform for Identifying Synthetic Promoters with Enhanced Cell-State Specificity (SPECS)

Wu et al.

Supplementary Figure 1

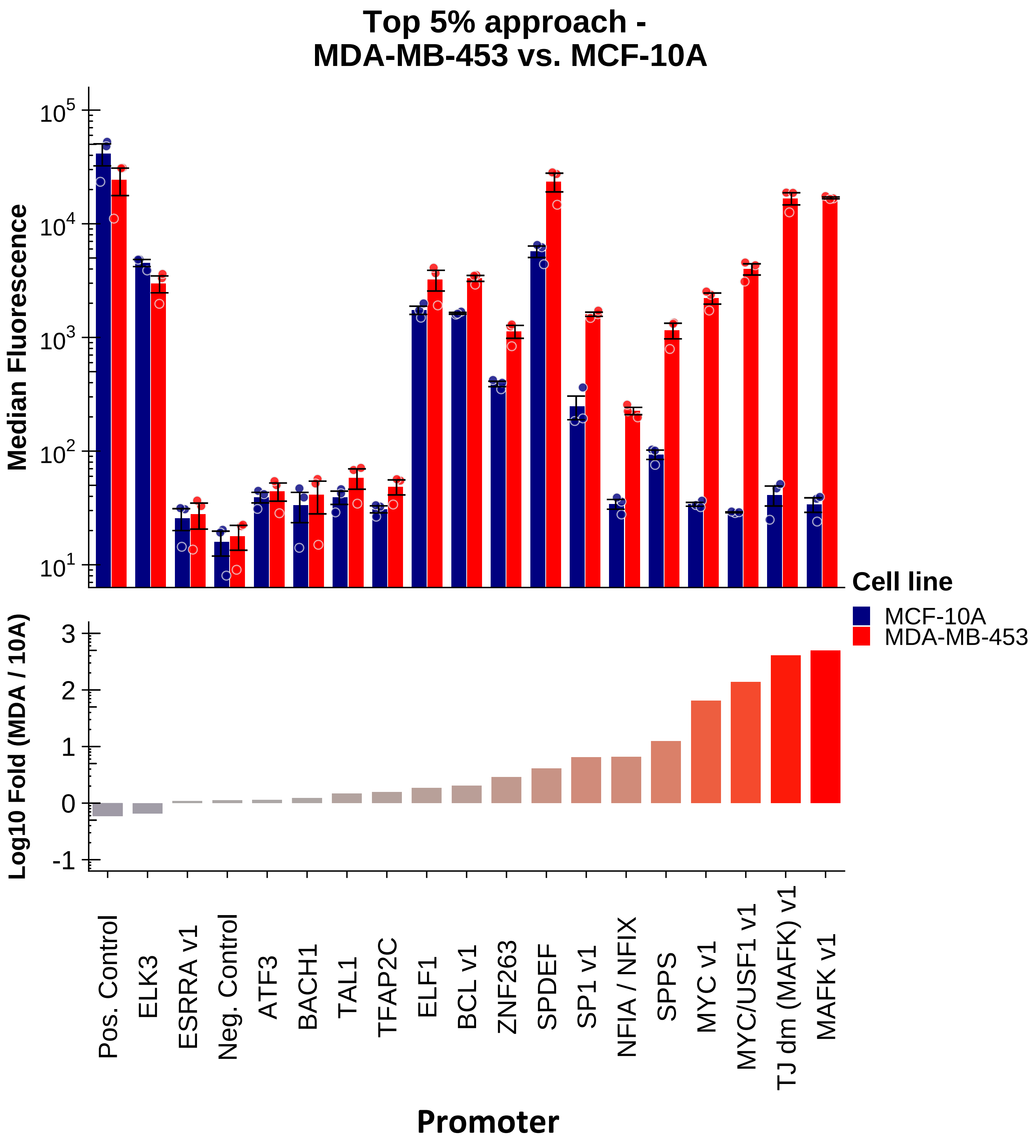

Supplementary Figure 1. The activities of promoters identified by the Top 5% approach. Promoters identified by the Top 5% approach presented up to ~500-fold activity difference between the breast cancer cell line MDA-MB-453 (MDA) and the normal breast cell line MCF-10A (10A). Some of these identified promoters were highly active and achieved median fluorescence intensities comparable to that seen with a constitutive Ubiquitin C promoter (Pos. Control). The negative control sample (Neg. Control) consisted of cells infected with a non-fluorescent protein. The dots represent the values of three biological replicates. Error bars represent S.E.M., N = 3 biological replicates. Source data are provided as a Source Data file.

Supplementary Figure 2

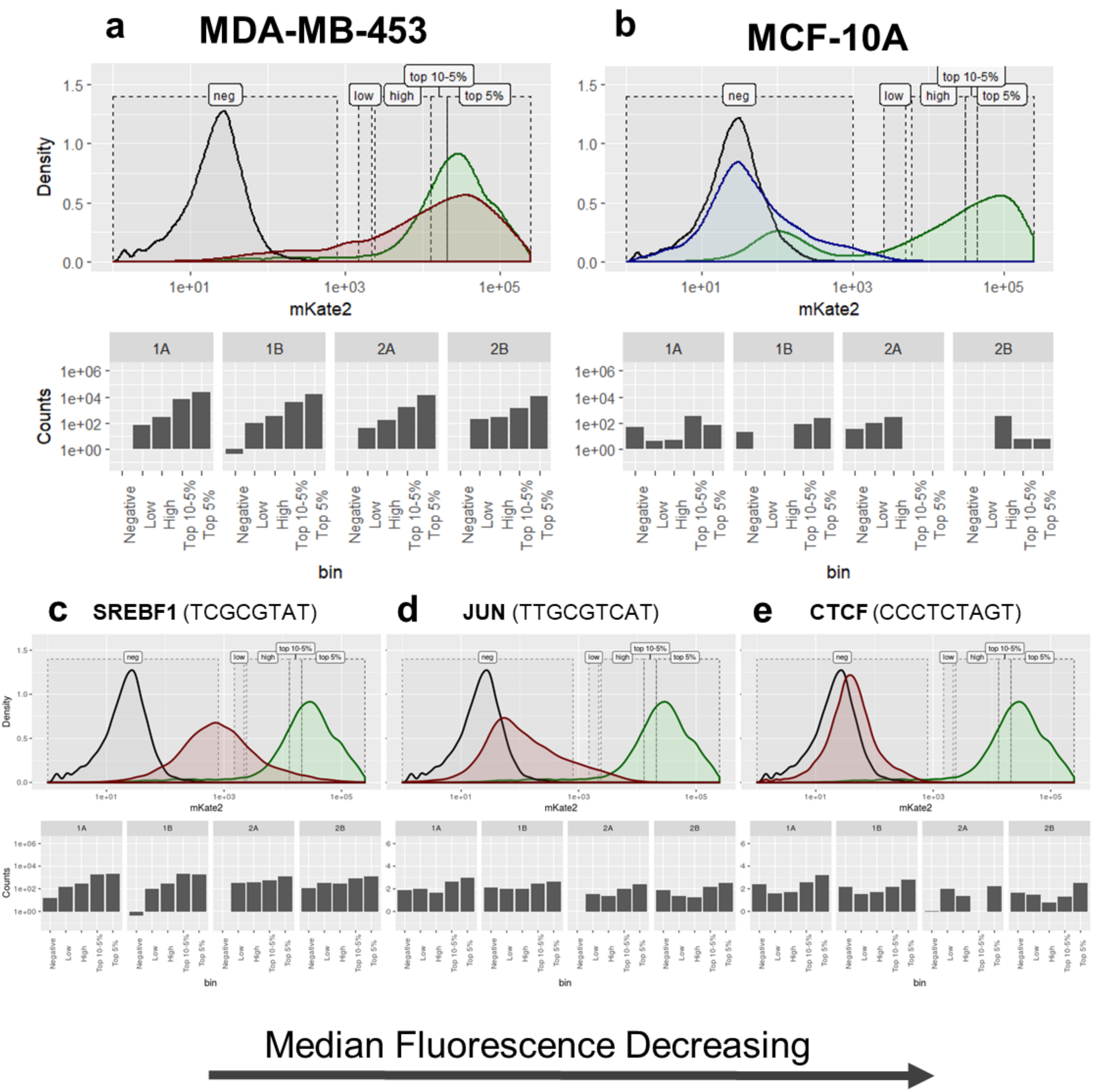

Supplementary Figure 2. Relationship between fluorescence and NGS read counts. (a) Comparison of the fluorescence distribution and NGS normalized counts of a promoter that contains the MAFK V1 TF-BS– (TGCTGAGTCAGCA) from the Top 5% shotgun cloning approach. This promoter exhibited very high activity in MDA-MB-453 cells and very low activity in MCF-10A cells. Dashed boxes represent the FACS gate for each bin. The numbers 1 and 2 denote data from 2 independent screening experiments. The letters A and B denote PCR technical replicates amplified from the promoter locus from genomic DNA for NGS. We observed that the fluorescence distribution of this promoter in MDA-MB-453 (red line) was comparable to that of the positive control UbC promoter (green line) and was much higher than that of the negative control sample (grey line). There were much higher counts in the positive bins than in the negative bin, with the highest counts being in the top 5% bin. **(b)** On the contrary, the fluorescence distribution of the same promoter in MCF-10A cells (blue line) was similar to that of the negative control sample (grey line) and much lower than that of the positive control sample (green line). **(c-e)** When three promoters with decreasing fluorescence intensities in MDA-MB-453 cells were compared (from Supplementary Fig. 2c to 2d to 2e; red lines in these 3 panels), there was a trend of decreasing total counts and a more uniform distribution of counts. A shift of counts from positive fluorescence bins to lower fluorescence or negative bins was also observed. For all panels, the negative control sample (grey line) consisted of cells infected with a non-fluorescent protein, and the positive control sample (green line) consisted of cells infected with a Ubiquitin C promoter expressing mKate2.

Supplementary Figure 3

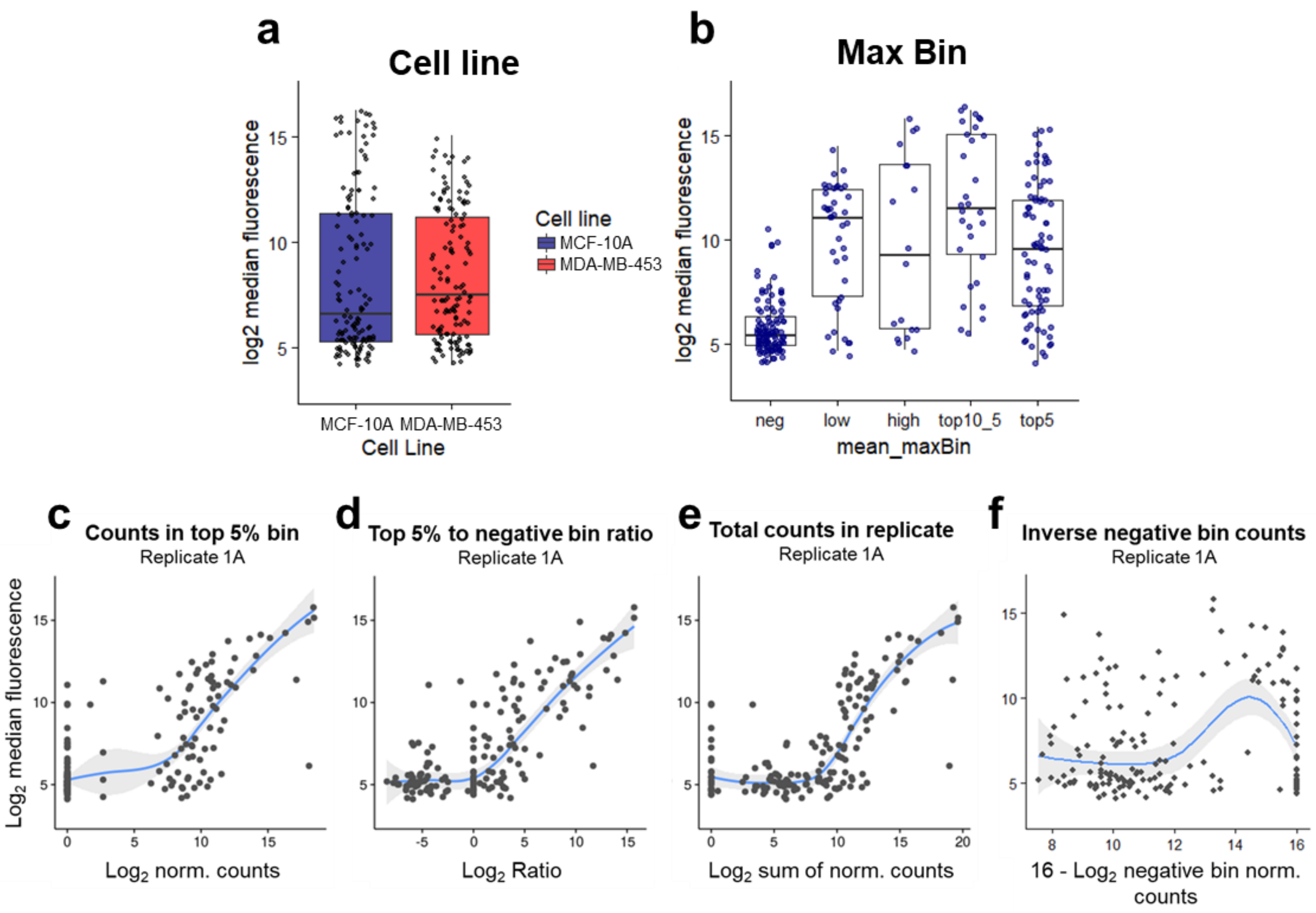

Supplementary Figure 3. Machine-learning features. (a) In the model, the type of cell line is used as a categorical feature. As seen by the median, MDA-MB-453 cells had a greater fluorescence than MCF-10A cells; this difference contributed to model predictions. Each dot represents the fluorescence intensity of an experimentally tested promoter. **(b)** Maximal Bin (Max Bin), the bin having the most counts for a specific promoter, is another categorical feature. The negative and top 5% bins contained the most promoters tested, with the top 5% showing greater fluorescence by the median than the negative bin. All other bins contained much fewer promoters tested. Each dot represents the fluorescence intensity of an experimentally tested promoter. For **(a)** and **(b)**, the boxes denote the lower quartile, the median, and the upper quartile. Whiskers denote the minimum and maximum up to 1.5x interquartile range. **(c-f)** Feature values are shown for continuous features (X-axis) plotted against $\log_2$ observed median fluorescence (Y-axis) for all validated promoters. Each dot represents the data from a validated promoter. Features include: $\log_2$value of the normalized counts of promoters in the top 5% bin (**c**); count ratio of the promoters in the top 5% bin to the negative bin **(d)**; total normalized counts for the promoters from all the biological and technical replicates **(e)**; and the "reverse" (16 (max) – $\log_2$counts) of the negative bin counts **(f)**. These features show a monotonically increasing approximation for fluorescence, with counts in the negative bin showing an inverse relationship. A description of the axes is provided in each subplot, and the blue line is the loess regression with the grey area being the 95% confidence interval. Feature data were displayed for all experimentally tested promoters.

Supplementary Figure 4

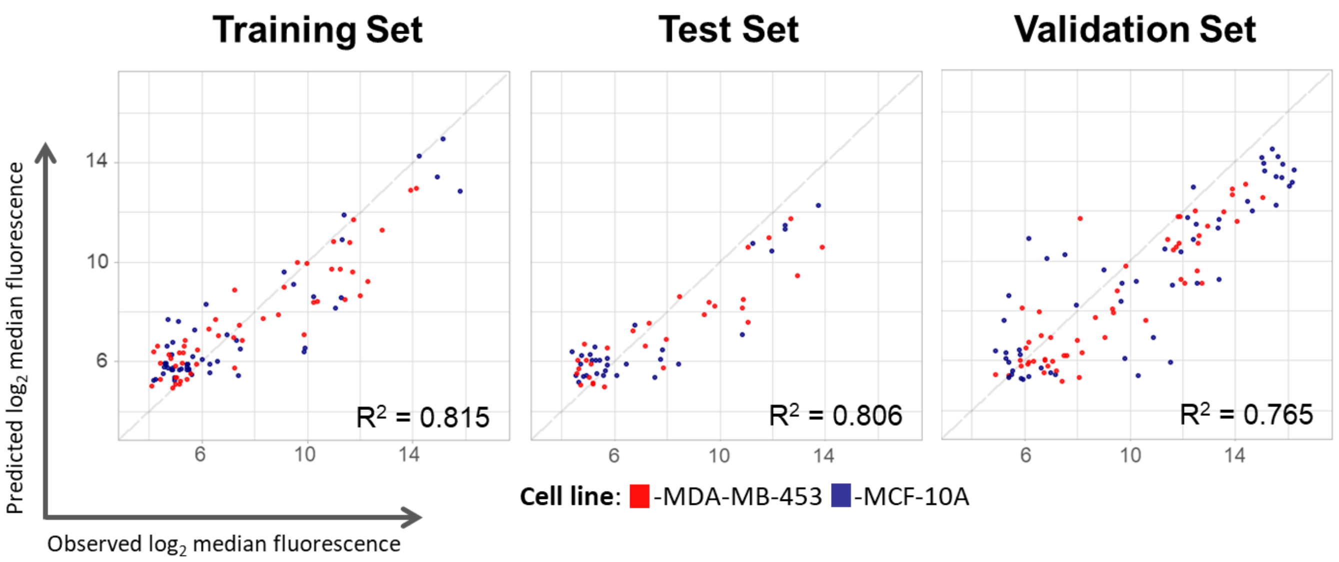

Supplementary Figure 4. Observed vs. predicted fluorescence for the 1st round of the machine learning predictions. Observed fluorescence data were compared with the fluorescence predicted by the GLMNET-inter model from the 1st round of machine learning prediction. Training (left) and test (middle) sets achieved comparable R^2 values, signaling that the model did not overfit the data. When the model was tested on the validation set (right), it performed less well for the mid-range of fluorescence (~2^8 - 2^{12}), especially in MCF-10A cells. The X-axis denotes the observed $\log_2$ median fluorescence; the Y-axis denotes the predicted $\log_2$ median fluorescence (this GLMNET-inter model was trained with data from 81 promoters: 17 from the Top 5% approach and 64 selected from the activity score metrics). The validation set consisted of an additional 54 promoters selected from the prediction result from the machine learning algorithm (GLMNET-inter = generalized linear model with elastic net regularization, using features and interaction terms between features).

Supplementary Figure 5

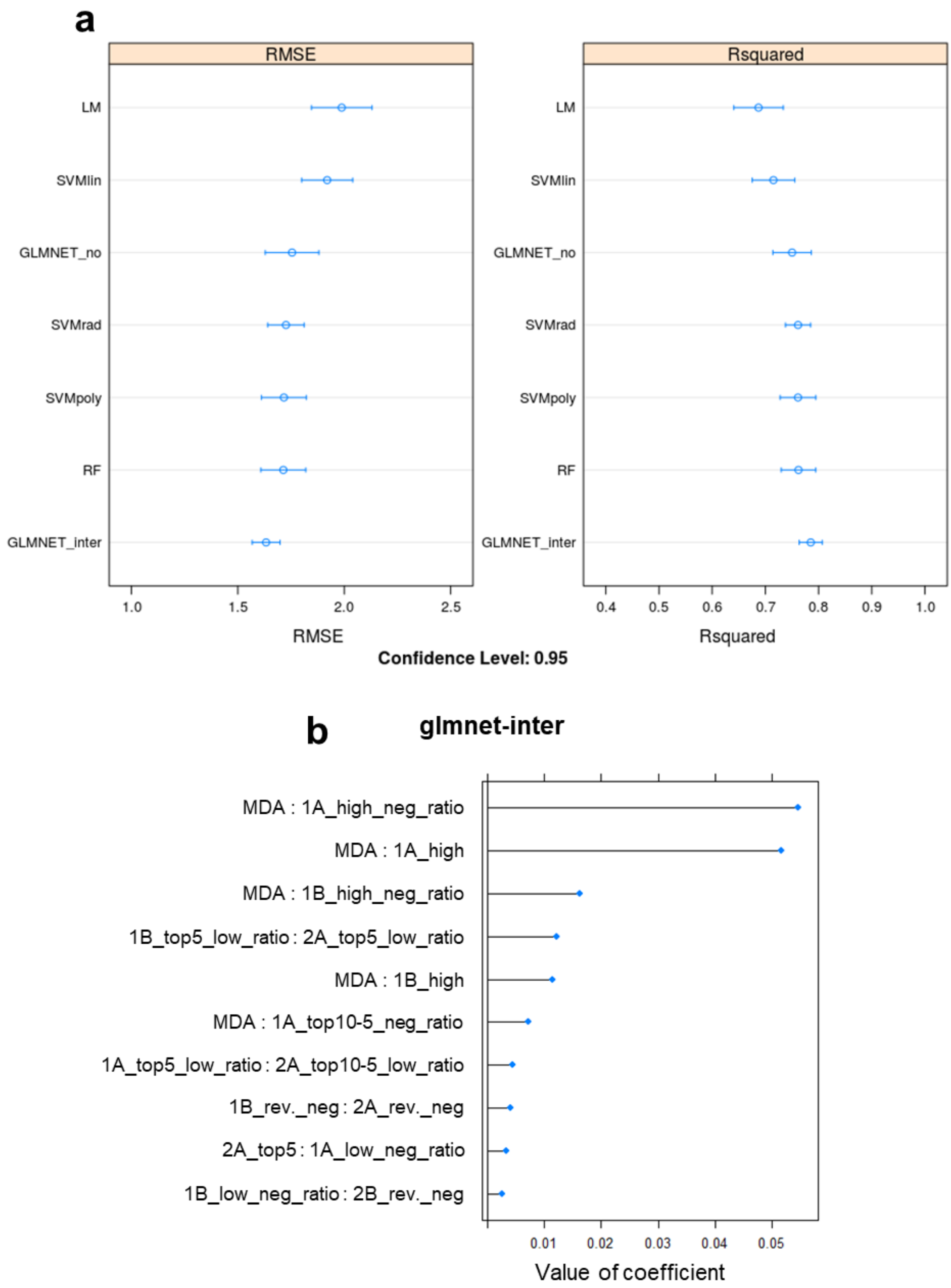

Supplementary Figure 5. Model performance and feature importance for the 2nd round of the machine learning predictions. (a) Models were compared based on RMSE and R-squared (R^2). Most of the models performed similarly with an R^2 of ~0.75 and RMSE of ~1.6-1.8 (with standard deviation of the data being 3.4), except for LM and SVMLin, which did not perform quite as well. GLMNET-inter was the best model, having a slight margin of performance over the others. Data presented in the plot contains the distribution of summary function (RMSE or R^2) for all resamples done for the model in the repeated CV when predicting the portion of the data which was left out. Model abbreviations: LM – linear regression; SVMLin – SVM with a linear kernel; GLMNET_no – generalized linear model with elastic net regularization (GLMNET) with no interaction terms; SVMRad – SVM with a radial kernel; SVMPoly – SVM with a polynomial kernel; RF – random forest regression; GLMNET_inter – GLMNET using features and interaction terms between the features; RMSE – root mean squared error, R^2 – Pearson's correlation coefficient. Error bars represent 95% CI. (**b**) Examining the coefficients of the features in the GLMNET-inter model, we identified as the most important features as: cell line (being MDA-MB-453), counts in high ("high") and top 5% bins ("top5"), high to negative bin ratio ("high_neg_ratio"), top 5% to low ratio ("top5_low_ratio"), or top 10%-5% to low ratio ("top10-5_low_ratio"), and the inverse negative ("rev._neg"). 1A-2B represent biological and technical replicates with the numbers denoting the biological replicates and the alphabets denoting the technical replicates.

Supplementary Table 1

		Predicted		Meassured		Fold	
Class	TFBS	MDA-453	MCF-10A	MDA-453	MCF-10A	MDA-453	MCF-10A
10A Spec.	RELA v6	41	22735	76	77233	0.00	1021.15
10A Spec.	RELA v7	66	13123	78	67400	0.00	858.96
10A Spec.	RELA v4	80	19205	119	70800	0.00	593.79
10A Spec.	RELA v8	50	13020	55	29367	0.00	538.51
10A Spec.	RELA v3	32	14349	123	66200	0.00	538.21
10A Spec.	RELA v9	40	36110	116	56500	0.00	488.47
10A Spec.	RELA v5	38	21284	144	54633	0.00	380.63
10A Spec.	NFKB1 v1	48	23583	114	39433	0.00	345.30
10A Spec.	NFKB v2	61	38401	103	33867	0.00	329.34
10A Spec.	NFKB1 v2	41	28701	331	70633	0.00	213.61
10A Spec.	NFKB v3	69	33599	254	50067	0.01	197.37
10A Spec.	NFKB v1	18	6315	83	8772	0.01	105.85
MDA Spec.	NFY	2640	28	3507	1227	2.86	0.35
MDA Spec.	SPDEF mm v2	5137	31	13749	1202	11.44	0.09
MDA Spec.	FOXA	4134	29	597	25	23.52	0.04
MDA Spec.	SPDEF mm v3	5262	48	32467	979	33.15	0.03
MDA Spec.	MYC v7	4764	41	3534	55	63.79	0.02
MDA Spec.	E2F1/E2F4	5038	51	3858	42	91.27	0.01
MDA Spec.	MYCN mm	5952	31	5700	55	103.64	0.01
MDA Spec.	FOXA3	3059	17	5043	44	114.61	0.01
MDA Spec.	MYC v6	4076	63	6508	49	133.63	0.01
MDA Spec.	MYC/USF1 v2	3091	22	6630	41	161.71	0.01
MDA Spec.	TJ dm (MAFK) v2	7016	25	28533	61	468.27	0.00
MDA Spec.	MAFK v3	2750	27	31133	39	794.89	0.00
Control	Neg. Control			19	27	0.71	1.40
Control	Pos. Control			37100	51667	0.72	1.39

Supplementary Table 1. Cell state-specific promoters derived from the validation set. Of the 54 promoters from the validation set, 12 were predicted to be MDA-MB-453 specific and 12 to be MCF-10A specific. Of the 12 predicted to be MDA-MB- 453 specific, 11 were indeed specific (fold > 10X). For MCF-10A, 12/12 were specific. Predictions are derived from the GLMNET-inter trained on the 1st set (81 promoters) only. Observed values are the average of median fluorescence in biological triplicates. Fold value is the activity fold-difference between the predicted cell state of interest / the other cell state.

Supplementary Note 1 - Features for machine learning

Features were engineered according to the observed relationship between log2 transformed counts and fluorescence. The numeric features included: counts (in each bin and replicate), ratio of bin X to negative (for each replicate), ratio of top 5-10% to low (for each replicate), ratio of top 5% to low (for each replicate), geometric mean of bin counts in replicates (for each bin), reverse negative counts (for each bin, using 16 - neg. where $2^{\wedge 16}$ is above the range measured for the FACS data), total replicate counts (for each bin) and total counts (in all replicates and bins). The categorical features included: cell line identity (MDA-MB-453 or MCF-10A) and maximum bin (negative, low, high, top 5-10%, top 5%) (Supplementary Fig. 3). Interaction terms among all features were also used in some of the models using R formula interface (as '*'). In the chosen model (GLMNET-inter), coefficients are regularized, and this can be thought of as an automatic feature selection [1].

Supplementary Note 2 – Identification of GSC-specific promoters

Due to low coverage of the library in the MGG4 experiment, the machine learning model described above was not deployable. Instead we sought to utilize the most important features identified by the model for manually prioritizing promoters. Some of the more important features were still calculable (for feature importance, see Supplementary Fig. 5), including total counts, counts in negative bin, and the bin with the most counts (i.e., most counts in negative bin). Thus, these features were used to rank the promoters and manually identify promising candidates ranking high on these metrics and showing reasonable coverage.

Supplementary References

1 Friedman, J., Hastie, T. & Tibshirani, R. Regularization Paths for Generalized Linear Models via Coordinate Descent. *J Stat Softw* **33**, 1-22 (2010).
