# Supplementary material for: A high-throughput screening and computation platform for identifying synthetic promoters with enhanced cell-state specificity (SPECS)
Source: Nat Commun. 2019 Jun 28;10:2880. doi: 10.1038/s41467-019-10912-8 (PMC6599391; doi:10.1038/s41467-019-10912-8)
Supplement: Supplementary file 2 — Reporting Summary [file 41467_2019_10912_MOESM2_ESM.pdf]

nature research

Corresponding author(s): Yuval Tabach; Timothy K. Lu

Last updated by author(s): 2019/5/16

Reporting Summary

Nature Research wishes to improve the reproducibility of the work that we publish. This form provides structure for consistency and transparency in reporting. For further information on Nature Research policies, see Authors & Referees and the Editorial Policy Checklist.

Statistics

For all statistical analyses, confirm that the following items are present in the figure legend, table legend, main text, or Methods section.

n/a	Confirmed	
☐	☒	The exact sample size (*n*) for each experimental group/condition, given as a discrete number and unit of measurement
☐	☒	A statement on whether measurements were taken from distinct samples or whether the same sample was measured repeatedly
☐	☒	The statistical test(s) used AND whether they are one- or two-sided<br>*Only common tests should be described solely by name; describe more complex techniques in the Methods section.*
☒	☐	A description of all covariates tested
☒	☐	A description of any assumptions or corrections, such as tests of normality and adjustment for multiple comparisons
☒	☐	A full description of the statistical parameters including central tendency (e.g. means) or other basic estimates (e.g. regression coefficient) AND variation (e.g. standard deviation) or associated estimates of uncertainty (e.g. confidence intervals)
☒	☐	For null hypothesis testing, the test statistic (e.g. *F*, *t*, *r*) with confidence intervals, effect sizes, degrees of freedom and *P* value noted<br>*Give P values as exact values whenever suitable.*
☒	☐	For Bayesian analysis, information on the choice of priors and Markov chain Monte Carlo settings
☒	☐	For hierarchical and complex designs, identification of the appropriate level for tests and full reporting of outcomes
☐	☒	Estimates of effect sizes (e.g. Cohen's *d*, Pearson's *r*), indicating how they were calculated

Our web collection on statistics for biologists contains articles on many of the points above.

Software and code

Policy information about availability of computer code

Data collection	Counts from next generation sequencing were analyzed using FastQC v0.11.5, FASTX-Toolkit 0.0.14, Bowtie2 v2.2.9, SAMtools. Counts were then processed by a custom R (v3.4.1) script and normalized using DESeq2.<br>The codes relevant to the analysis of this study are available at GitHub: https://github.com/dst1/SPECS.
Data analysis	Flow cytometry data was analyzed using flowCore in R and FlowJo (FlowJo LLC) version 7.6.1. Machine learning models were produced using the caret R package.<br>The codes relevant to the analysis of this study are available at GitHub: https://github.com/dst1/SPECS.

For manuscripts utilizing custom algorithms or software that are central to the research but not yet described in published literature, software must be made available to editors/reviewers. We strongly encourage code deposition in a community repository (e.g. GitHub). See the Nature Research guidelines for submitting code & software for further information.

Data

Policy information about availability of data

All manuscripts must include a data availability statement. This statement should provide the following information, where applicable:

- Accession codes, unique identifiers, or web links for publicly available datasets
- A list of figures that have associated raw data
- A description of any restrictions on data availability

The data that support the findings of this study are available from the authors on reasonable request.

Field-specific reporting

Please select the one below that is the best fit for your research. If you are not sure, read the appropriate sections before making your selection.

☒ Life sciences ☐ Behavioural & social sciences ☐ Ecological, evolutionary & environmental sciences

For a reference copy of the document with all sections, see nature.com/documents/nr-reporting-summary-flat.pdf

Life sciences study design

All studies must disclose on these points even when the disclosure is negative.

Sample size	At least three biological replicates were performed for each promoter validation experiment (N = 3). From prior studies and the expected experimental variance, we determined this sample size to be sufficient to ensure reproducibility.
Data exclusions	No data were excluded.
Replication	The library screens were done with biological replicates and PCR technical replicates. The promoter validation experiments were done with biological replicates (N = 3).
Randomization	For the Top 5 % approach, random colonies were picked and tested from the transformation plates.
Blinding	No blinding was performed.

Reporting for specific materials, systems and methods

We require information from authors about some types of materials, experimental systems and methods used in many studies. Here, indicate whether each material, system or method listed is relevant to your study. If you are not sure if a list item applies to your research, read the appropriate section before selecting a response.

Materials & experimental systems

n/a	Involved in the study	
☒	☐	Antibodies
☐	☒	Eukaryotic cell lines
☒	☐	Palaeontology
☒	☐	Animals and other organisms
☒	☐	Human research participants
☒	☐	Clinical data

Methods

n/a	Involved in the study	
☒	☐	ChIP-seq
☐	☒	Flow cytometry
☒	☐	MRI-based neuroimaging

Eukaryotic cell lines

Policy information about cell lines

Cell line source(s)	All human cell lines except iPSC and MGG4 were obtained from ATCC. iPSC cell line was obtained from Ron Weiss lab at MIT and MGG4 cell line was obtained from Samuel Rabkin lab at MGH.
Authentication	Human cell lines from ATCC were not authenticated because these cell lines were rigorously authenticated by ATCC. All cell lines were recently obtained from ATCC or collaborators and were expanded and frozen at very low passages.
Mycoplasma contamination	Cell lines were tested negative for mycoplasma by Division of Comparative Medicine at MIT.
Commonly misidentified lines (See ICLAC register)	No commonly misidentified lines were used in this study.

Flow Cytometry

Plots

Confirm that:

- [ ] The axis labels state the marker and fluorochrome used (e.g. CD4-FITC).
- [ ] The axis scales are clearly visible. Include numbers along axes only for bottom left plot of group (a 'group' is an analysis of identical markers).
- [ ] All plots are contour plots with outliers or pseudocolor plots.
- [ ] A numerical value for number of cells or percentage (with statistics) is provided.

Methodology

Sample preparation	All cells were trypsinized or dissociated to single cells, filtered through 0.22um filter, and run through flow cytometer.
Instrument	BD Biosciences LSRII Fortessa cytometer
Software	BD Biosciences FACSDiva
Cell population abundance	All cell populations analyzed by the flow cytometer are highly abundant pure populations of cultured cell lines.
Gating strategy	Cells were first gated according to FSC-A and SSC-A parameters to identify the major viable population (generally > 90% of the cell population), followed by the measurement of the fluorescence intensity.

- [ ] Tick this box to confirm that a figure exemplifying the gating strategy is provided in the Supplementary Information.
